# The association between multimorbidity and mobility disability-free life expectancy in adults aged 85 years and over: A modelling study in the Newcastle 85+ cohort

**DOI:** 10.1371/journal.pmed.1004130

**Published:** 2022-11-14

**Authors:** Laurie E. Davies, Stewart W. Mercer, Katie Brittain, Carol Jagger, Louise Robinson, Andrew Kingston

**Affiliations:** 1 Population Health Sciences Institute, Newcastle University, Newcastle upon Tyne, United Kingdom; 2 Advanced Care Research Centre, Usher Institute, University of Edinburgh, Edinburgh, United Kingdom; Harvard Medical School, UNITED STATES

## Abstract

**Background:**

Mobility disability is predictive of further functional decline and can itself compromise older people’s capacity (and preference) to live independently. The world’s population is also ageing, and multimorbidity is the norm in those aged ≥85. What is unclear in this age group, is the influence of multimorbidity on (a) transitions in mobility disability and (b) mobility disability-free life expectancy (mobDFLE).

**Methods and findings:**

Using multistate modelling in an inception cohort of 714 85-year-olds followed over a 10-year period (aged 85 in 2006 to 95 in 2016), we investigated the association between increasing numbers of long-term conditions and (1) mobility disability incidence, (2) recovery from mobility disability and (3) death, and then explored how this shaped the remaining life expectancy free from mobility disability at age 85. Models were adjusted for age, sex, disease group count, BMI and education. We defined mobility disability based on participants’ self-reported ability to get around the house, go up and down stairs/steps, and walk at least 400 yards; participants were defined as having mobility disability if, for one or more these activities, they had any difficulty with them or could not perform them. Data were drawn from the Newcastle 85+ Study: a longitudinal population-based cohort study that recruited community-dwelling and institutionalised individuals from Newcastle upon Tyne and North Tyneside general practices.

We observed that each additional disease was associated with a 16% increased risk of incident mobility disability (hazard ratio (HR) 1.16, 95% confidence interval (CI): 1.07 to 1.25, *p* < 0.001), a 26% decrease in the chance of recovery from this state (HR 0.74, 95% CI: 0.63 to 0.86, *p* < 0.001), and a 12% increased risk of death with mobility disability (HR: 1.12, 95% CI: 1.07- to .17, *p* < 0.001). This translated to reductions in mobDFLE with increasing numbers of long-term conditions. However, residual and unmeasured confounding cannot be excluded from these analyses, and there may have been unobserved transitions to/from mobility disability between interviews and prior to death.

**Conclusions:**

We suggest 2 implications from this work. (1) Our findings support calls for a greater focus on the prevention of multimorbidity as populations age. (2) As more time spent with mobility disability could potentially lead to greater care needs, maintaining independence with increasing age should also be a key focus for health/social care and reablement services.

## Abstract 2

In a modelling study from Newcastle, Dr Laurie Davies and colleagues explore the association between multimorbidity and mobility disability-free life expectancy in adults aged 85 years and over.

## Author summary

**Why was this study done?:**

- Multimorbidity is the norm in growing older populations.
- Mobility disability also has profound consequences for health, well-being and independent living.
- However, there is a dearth of research exploring the relationship between multimorbidity and mobility disability in those aged ≥85, even though attention is now more focussed on the quality of remaining life expectancy.

**What did the researchers do and find?:**

- In an inception cohort of 85-year-olds followed over 10 years (age 85 to 95), we explored the association between multimorbidity and transitions in mobility disability, and then examined how this was associated with mobility disability-free life expectancy (mobDFLE).
- We found that there is no threshold beyond which multimorbidity becomes disabling in those aged ≥85; rather, each additional disease is associated with a 16% increased risk of incident mobility disability.
- This translates to reductions in mobDFLE with increasing numbers of long-term conditions.

**What do these findings mean?:**

- Our findings suggest that, in those aged ≥85, multimorbidity is an important determinant of mobility disability and the number of years spent living with it.
- As mobility disability can lead to greater care needs, preventing multimorbidity and maintaining independence including from earlier in the life course could be beneficial.

## Introduction

The World Health Organisation prioritises the preservation of functional ability to enable older people to carry on doing the things in life to which they attribute value [1], like the shopping and the housework, the ability to go outdoors and meet other people [2]. This priority complements the United Kingdom Ageing Society Grand Challenge, which aims to “ensure that people can enjoy at least 5 extra healthy, independent years of life by 2035, while narrowing the gap between the experience of the richest and poorest” [3]. The significance of these goals reflects the profound impact that loss of functional ability can have on quality of life, its power to reinforce further functional decline, the complex bidirectional interplay with diseases, the increased risk for medical and social care, and its association with mortality [4].

Functional ability is generally measured through activities that we do every day to maintain independence, such as walking, washing and eating. Losing the capacity to carry out such tasks leads to disability and when this happens, an underlying hierarchical property of the disability process is revealed [5]. Disability onset usually occurs first with mobility (e.g., walking and using steps); mobility disability then predicts the incidence of disability with tasks essential to living (e.g., meal preparation, housework) and the ability to care for oneself (e.g., dressing and using the bathroom) [5,6]. Mobility disability therefore represents the gateway to further functional decline, and can itself compromise older people’s capacity (and preference) to live independently [7]. However, the factors that drive the incidence of mobility disability are less well described, despite it also being the optimal point for interventions to slow down functional decline and/or regain independence [8].

For those aged ≥85 years, who are the fastest growing age group in many high-income countries [9], the identification of disease-based factors that increase the risk of mobility disability is clouded by their chronic co-occurrence, i.e., multimorbidity [10]. In addition, we do not know how, as the number of multiple long-term conditions increase, this impacts mobility disability incidence, or recovery from mobility disability, or the amount of remaining life expectancy a person aged 85 may expect to spend free of mobility disability. Furthermore, the age at which diseases occur, and their type, are modified by factors related to lifestyle and sociodemographics [11].

Through multistate modelling in an inception cohort of 85-year-olds followed over 10 years (age 85 to 95 years), we aimed to examine the association between increasing numbers of long-term conditions and (i) mobility disability incidence, (ii) recovery from mobility disability and (iii) death, and (iv) then explore how this shapes mobility disability-free life expectancy (mobDFLE), the remaining life expectancy free from mobility disability at age 85.

## Methods

This study is reported as per the Strengthening the Reporting of Observational Studies in Epidemiology (STROBE) guideline (S1 Appendix).

### Participants

The Newcastle 85+ Study is a population-based longitudinal study of community-dwelling and institutionalised individuals who were born in 1921, aged 85 in 2006, and permanently registered with one of 53 participating general practices in Newcastle or North Tyneside [12]. When the study began (2006), participants were broadly representative of 85-year-olds in England and Wales in terms of sex, care home residence and whether living alone, but participants with end-stage terminal illness were excluded (*n* = 11) [13]. Data were gathered by 2 methods: (i) multidimensional health assessment by a trained research nurse in the participant’s place of residence, inclusive of care homes, at baseline (wave 1), 18 months (wave 2), 36 months (wave 3), 60 months (wave 4) and 120 months (wave 5), and (ii) review of general practice medical records at baseline, waves 3, 4 and 5 [14]. Participants received the same assessment at baseline and follow-up to look for changes in mobility disability items. Full details of the study design, participant recruitment and representativeness are reported elsewhere [12–14]. Further details, including study questionnaires and the GP record review proforma, can be found on the Newcastle 85+ Study website https://research.ncl.ac.uk/85plus/, while study retention can be found in S2 Appendix. Of the 849 people who were eligible for analyses at baseline (S2 Appendix), we constructed a measure of mobility disability on 845 individuals (524 females and 321 males), of whom, 714 (424 females and 290 males) had complete data for all confounding variables used in the analysis. Over the 5 waves of data collection, participants were lost to follow-up for health reasons, nonhealth reasons and death [15].

### Ethical approval

The Newcastle 85+ Study was approved by the Newcastle and North Tyneside Local Research Committee One (Ref: 06/Q0905/2). Written informed consent was obtained from participants, and where people lacked capacity to consent—for example, because of dementia—an opinion was sought from a relative or carer (a “consultee”) [13].

### Definition of mobility disability

Using items predominantly from the Groningen Activity Restriction Scale [16] as previously described [17,18], a binary variable for mobility disability was constructed based on participants self-reported ability to get around the house, go up and down stairs/steps and walk at least 400 yards [17,18]. Participants were defined as having mobility disability if, for one or more these activities, they had any difficulty with them (responding yes to “I have some difficulty doing this by myself,” or “I can only do this by myself if I use an aid or appliance”) or could not perform them (responding yes to “I am unable to do this by myself, I need someone else’s help”). Data were gathered from questionnaires from the multidimensional health assessment.

### Definition of multiple long-term conditions

Disease group count was created by scoring 9 chronic diseases as either present (1) or absent (0), based on review of general practice medical records by trained research nurses (arthritis, diabetes, hypertension, cardiac disease, chronic obstructive pulmonary disease, other respiratory disease, stroke, other cerebrovascular disease and cancer in the past 5 years excluding nonmelanoma skin cancer). Some conditions were grouped into a category (e.g., all arthritic diseases) while others were retained as single entities (e.g., hypertension). Full details of disease status construction can be found in S3 Appendix.

### Other variables

Age, sex, years in education and body mass index (BMI), calculated as kg weight/m^2^ height and categorized as <18.5 (underweight), 18.5 to 24.99 (healthy weight), 25 to 29.99 (overweight) and ≥30 (obese) [19], were also included in the model building strategy. These data were obtained from general practice record review (age, sex) and a multidimensional health assessment comprising questionnaires (years in education) and measurement tests (BMI). The following sociodemographic variables, derived from multidimensional health assessment questionnaire data, were used to characterise the sample: housing (standard/sheltered/care home), living arrangements (alone/not alone), marital status (never married/married/divorced/separated or widowed) and socioeconomic position (<25th, 25th to 75th, and >75th centile Index of Multiple Deprivation) [20].

### Statistical analysis

**Fig 1:**
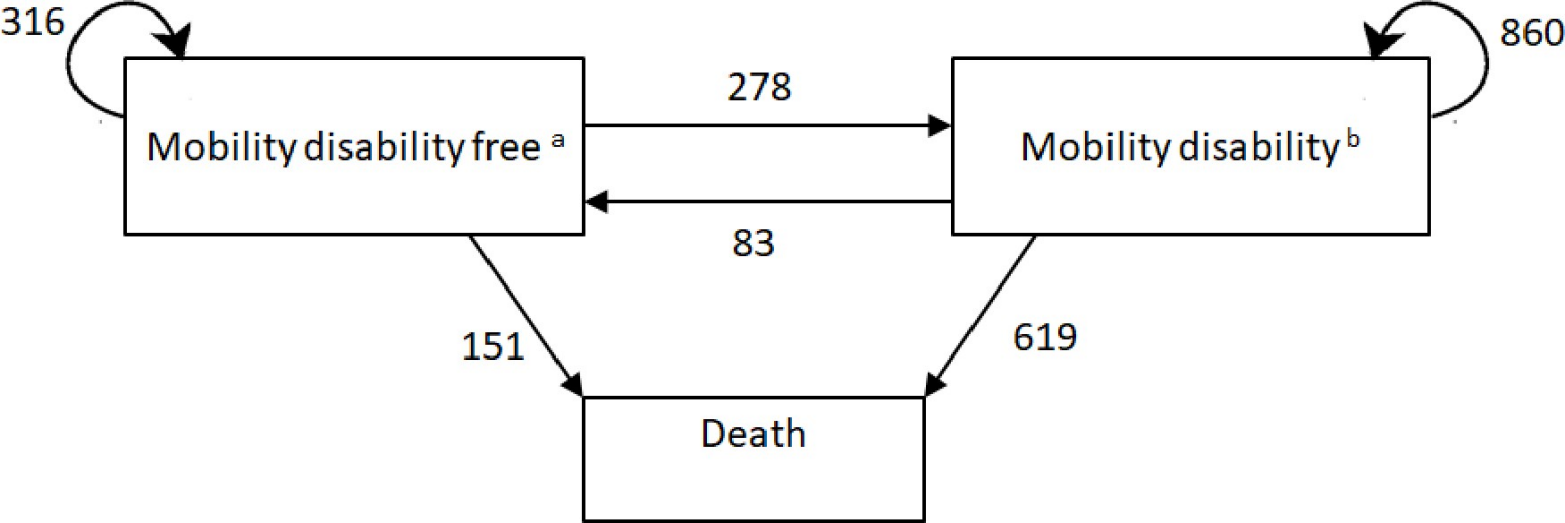
Markov multistate transition model for mobility disability-death in the Newcastle 85+ Study. ^a^Censored = 23; ^b^Censored = 53. Note: numbers represent the number of transitions between states, not the number of people that moved. For example, there were 83 transitions, classed as recovery, from the mobility disability to mobility disability-free state, while there were 316 transitions for remaining mobility disability free between the Newcastle 85+ Study waves and 860 transitions for remaining with mobility disability between the study waves.

The sociodemographic and health characteristics of the baseline cohort were examined through descriptive statistics. To model transitions to and from mobility disability, and to death in the inception cohort of 85-year-olds followed over 10 years (age 85 to 95 years), we fitted a Markov multistate transition model with 3 states—mobility disability free, mobility disability and death (Fig 1)—using a Gompertz model and the “msm” package [21]. Recovery (transitioning from mobility disability to mobility disability free) was defined as no longer having difficulty with any of the 3 mobility disability items. Survival time was calculated from the date of baseline interview to the date of death or censoring at 120 months (10 years from baseline or after final interview if a participant had taken part in the 10-year follow-up). Age was used as a time-dependent covariate under the Gompertz model to allow piecewise-constant approximation of the dependency on age [22]. Models were adjusted in stages as follows: age and disease group count (model 1); age, sex and disease group count (model 2); age, sex, disease group count and BMI (model 3); age, sex, disease group count, BMI and education (model 4). Using model 4 estimates, we implemented the ELECT library (estimating life expectancies for continuous time) to estimate state specific life expectancy, with 500 replications of the points estimates to approximate uncertainty [22]. Briefly, ELECT uses established methodology to calculate state specific life expectancies using numerical methods and the transition probabilities defined by the state space (the possible states and transitions) of a fitted multistate model [22,23]. For our estimates, we held education at mean years and BMI at normal weight, and for each disease group count, we calculated the remaining life expectancy with and without mobility disability in the male and female participants at age 85. All covariates (excepting fixed variables—sex and years in education) were treated as time-varying to account for their values potentially changing over time (for example, due to incident disease with respect to multiple long-term conditions).

We did not have a prospective analysis plan; our analysis was decided when our research question was formed, but we made 2 changes to it after peer review: (1) Upon investigating a wide confidence interval raised by 1 reviewer, we detected a small error in our analytical code, which we rectified. (2) We reanalysed our data with the ELECT library to estimate life expectancy, as in response to comments from reviewers and wider reading, we learnt that our previous approximation using mean sojourn times was not suitable [22]. Analyses were performed using R version 4.0.2.

## Results

### Participant characteristics

Of the 845 baseline participants (aged 85), most were female (62.01%, 524/845), educated for approximately 9 years (mean: 9.91, standard deviation: 1.86), lived in standard housing (76.6%, 647/845), lived alone (60.9%, 462/759), were widowed (58.9%, 495/841) and had multiple long-term conditions (mean disease group count: 3.22, standard deviation: 1.85). Approximately half of the participants belonged to the 25th to 75th centile Index of Multiple Deprivation (50.3%, 425/845), were of healthy weight (51.2%, 368/719) and had mobility disability (56.3%, 476/845) (Table 1). The characteristics of the baseline participants according to the number of disease groups are shown in S4 Appendix.

**Table 1:** Baseline sociodemographic and health characteristics of Newcastle 85+ participants.

	% of total (n)
**Sex**	100 (845)
Male	37.99 (321)
Female	62.01 (524)
**Education (years) (mean (SD))**	9.91 (1.86)
**Housing**	
Standard	76.57 (647)
Sheltered	13.37 (113)
Care home	10.06 (85)
**Living alone**	60.87 (462)
**Marital status**	
Never married	8.20 (69)
Married	30.20 (254)
Divorced/separated	2.73 (23)
Widowed	58.86 (495)
**Deprivation (IMD)**	
<25th centile	25.21 (213)
25th–75th centile	50.29 (425)
>75th centile	24.50 (207)
**BMI (kg/m** ^2^ **)**	
<18.5: underweight	6.54 (47)
18.5–24.99: healthy weight	51.18 (368)
25–29.99: overweight	32.82 (236)
≥30: obese	9.46 (68)
**Mobility disability**	56.33 (476)
**Disease group count (mean (SD))**	3.22 (1.85)

BMI, body mass index; IMD, Index of Multiple Deprivation; SD, standard deviation.

Where numbers do not add up to 845 data are missing.

### Mobility disability prevalence over 10 years (from age 85 to 95)

**Fig 2:**
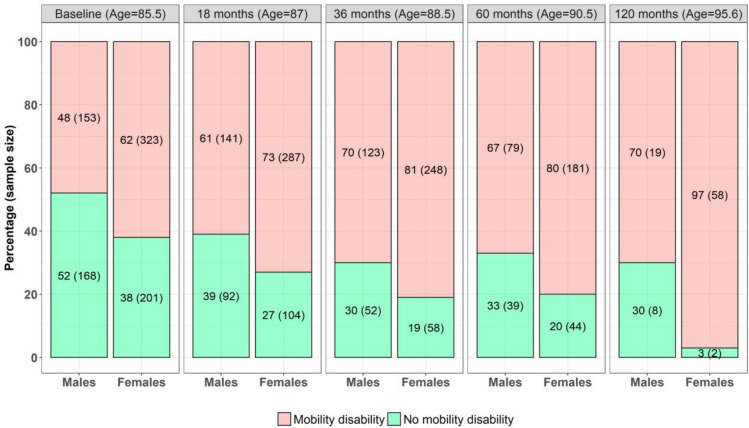
Prevalence of self-reported mobility disability in male and female participants from age 85–95. Note: ages represent mean ages.

The prevalence of mobility disability broadly increased in the female participants through to age 95 but plateaued in the male participants from 88 years of age (36 months) (Fig 2).

### Associations between sociodemographic/health factors and transitions between mobility disability states and death over 10 years

For each additional disease, the risk of incident mobility disability was increased by 16% (hazard ratio (HR) 1.16, 95% confidence interval (CI): 1.07 to 1.25, *p* < 0.001), the chance of recovery was reduced by 26% (HR 0.74, 95% CI: 0.63 to 0.86, *p* < 0.001), and the risk of death with mobility disability was increased by 12% (HR 1.12, 95% CI: 1.07 to 1.17, *p* < 0.001). Female participants had a higher risk of incident mobility disability than the male participants (HR: 1.64, 95% CI: 1.25 to 2.14, *p* < 0.001), and a lower risk of death with mobility disability (HR: 0.61, 95% CI: 0.52 to 0.72, *p* < 0.001). For every annual increase in age, the risk of death with mobility disability increased by 8% (HR: 1.08, 95% CI: 1.05 to 1.11, *p* < 0.001). Those overweight (BMI 25 to 29.99 kg/m^2^) were more likely to develop incident mobility disability than people of a healthy weight (HR: 1.51, 95% CI: 1.14 to 2.02, *p* < 0.05) (Table 2, Model 4, adjusted for disease group count, age, sex, BMI and years in education).

**Table 2:** Hazard ratios (HR) and 95% confidence intervals (95% CI) for transitions between mobility disability states and death.

	Model 1 HR (95% CI), *p*-value	Model 2 HR (95% CI), *p*-value	Model 3 HR (95% CI), *p*-value	Model 4 HR (95% CI), *p*-value
**Incident mobility disability**				
Disease group count	1.12 (1.04-1.22), *p* < 0.01	1.14 (1.06-1.24), *p* < 0.01	1.16 (1.07-1.25), *p* < 0.001	1.16 (1.07-1.25), *p* < 0.001
Age	1.01 (0.95-1.08), *p* = 0.77	1.01 (0.95-1.08), *p* = 0.17	1.02 (0.96-1.09), *p* = 0.55	1.02 (0.96-1.09), *p* = 0.55
Sex^a^	-	1.52 (1.18-1.95), *p* < 0.01	1.67 (1.28-2.18), *p* < 0.001	1.64 (1.25-2.14), *p* < 0.001
BMI (kg/m^2^)				
<18.5: underweight	-	-	0.97 (0.60-1.57), *p* = 0.91	0.98 (0.60-1.60), *p* = 0.94
18.5-24.99: healthy weight	-	-	Reference	Reference
25-29.99: overweight	-	-	1.51 (1.14-1.99), *p* < 0.05	1.51 (1.14-2.02), *p* < 0.05
**≥**30: obese	-	-	1.47 (0.86-2.50), *p* = 0.16	1.47 (0.86-2.52), *p* = 0.16
Education (years)	-	-	-	0.97 (0.82-1.14), *p* = 0.73
**Recovery from mobility disability**				
Disease group count	0.74 (0.64-0.86), *p* < 0.001	0.75 (0.64-0.86), *p* < 0.001	0.74 (0.64-0.86), *p* < 0.001	0.74 (0.63-0.86), *p* < 0.001
Age	0.87 (0.75-1.01), *p* = 0.07	0.86 (0.75-1.00), *p* = 0.04	0.87 (0.75-1.01), *p* = 0.07	0.87 (0.75-1.01), *p* = 0.07
Sex^a^	-	1.10 (0.67-1.80), *p* = 0.72	1.13 (0.69-1.86), *p* = 0.64	1.12 (0.68-1.85), *p* = 0.67
BMI (kg/m^2^)				
<18.5: underweight	-	-	0.58 (0.22-1.55), *p* = 0.28	0.57 (0.22-1.53), *p* = 0.26
18.5-24.99: healthy weight	-	-	Reference	Reference
25-29.99: overweight	-	-	1.51 (0.90-2.53), *p* = 0.12	1.55 (0.92-2.61), *p* = 0.10
≥30: obese	-	-	1.05 (0.40-2.75), *p* = 0.93	1.02 (0.38-2.71), *p* = 0.97
Education (years)	-	-	-	0.80 (0.56-1.13), *p* = 0.21
**Death with mobility disability**				
Disease group count	1.10 (1.06-1.15), *p* < 0.001	1.11 (1.06-1.15), *p* < 0.001	1.11 (1.07-1.16), *p* < 0.001	1.12 (1.07-1.17), *p* < 0.001
Age	1.07 (1.04-1.10), *p* < 0.001	1.07 (1.04-1.10), *p* < 0.001	1.07 (1.04-1.10), *p* < 0.001	1.08 (1.05-1.11), *p* < 0.001
Sex^a^	-	0.61 (0.52-0.71), *p* < 0.001	0.61 (0.52-0.72), *p* < 0.001	0.61 (0.52-0.72), *p* < 0.001
BMI (kg/m^2^)				
<18.5: underweight	-	-	1.11 (0.85-1.44), *p* = 0.45	1.14 (0.88-1.49), *p* = 0.33
18.5-24.99: healthy weight	-	-	Reference	Reference
25-29.99: overweight	-	-	0.80 (0.67-0.96), *p* < 0.05	0.81 (0.68-0.96), *p* < 0.05
≥30: obese	-	-	0.77 (0.59-1.01), *p* = 0.06	0.79 (0.60-1.04), *p* = 0.09
Education (years)	-	-	-	0.96 (0.87-1.07), *p* = 0.45
**Death without mobility disability**				
Disease group count	1.04 (0.71-1.52), *p* = 0.85	0.99 (0.69-1.42), *p* = 0.96	0.87 (0.62-1.24), *p* = 0.44	0.87 (0.62-1.23), *p* = 0.43
Age	0.71 (0.45-1.11), *p* = 0.13	0.68 (0.43-1.06), *p* = 0.09	0.59 (0.32-1.10), *p* = 0.09	0.60 (0.33-1.08), *p* = 0.09
Sex ^a^	-	0.67 (0.22-2.03), *p* = 0.49	0.41 (0.13-1.31), *p* = 0.13	0.42 (0.14-1.29), *p* = 0.13
BMI (kg/m^2^)				
<18.5: underweight	-	-	1.29 (0.20-8.49), *p* = 0.80	1.27 (0.20-8.06), *p* = 0.81
18.5-24.99: healthy weight	-	-	Reference	Reference
25-29.99: overweight	-	-	0.42 (0.10-1.73), *p* = 0.24	0.41 (0.09-1.81), *p* = 0.25
≥30: obese	-	-	0.74 (0.08-7.13), *p* = 0.80	0.73 (0.08-6.58), *p* = 0.79
Education (years)	-	-	-	0.86 (0.41-1.82), *p* = 0.70

^a^Male participants were the reference category.

BMI, body mass index; CI, confidence interval; HR, hazard ratio.

Note: Model 1 is adjusted for disease group count and age; Model 2 is adjusted for disease group count, age and sex; Model 3 is adjusted for disease group count, age, sex and BMI; Model 4 is adjusted for disease group count, age, sex, BMI and years in education.

### Association between multiple long-term conditions and mobility disability-free life expectancy in male and female participants at age 85 over 10 years

**Fig 3:**
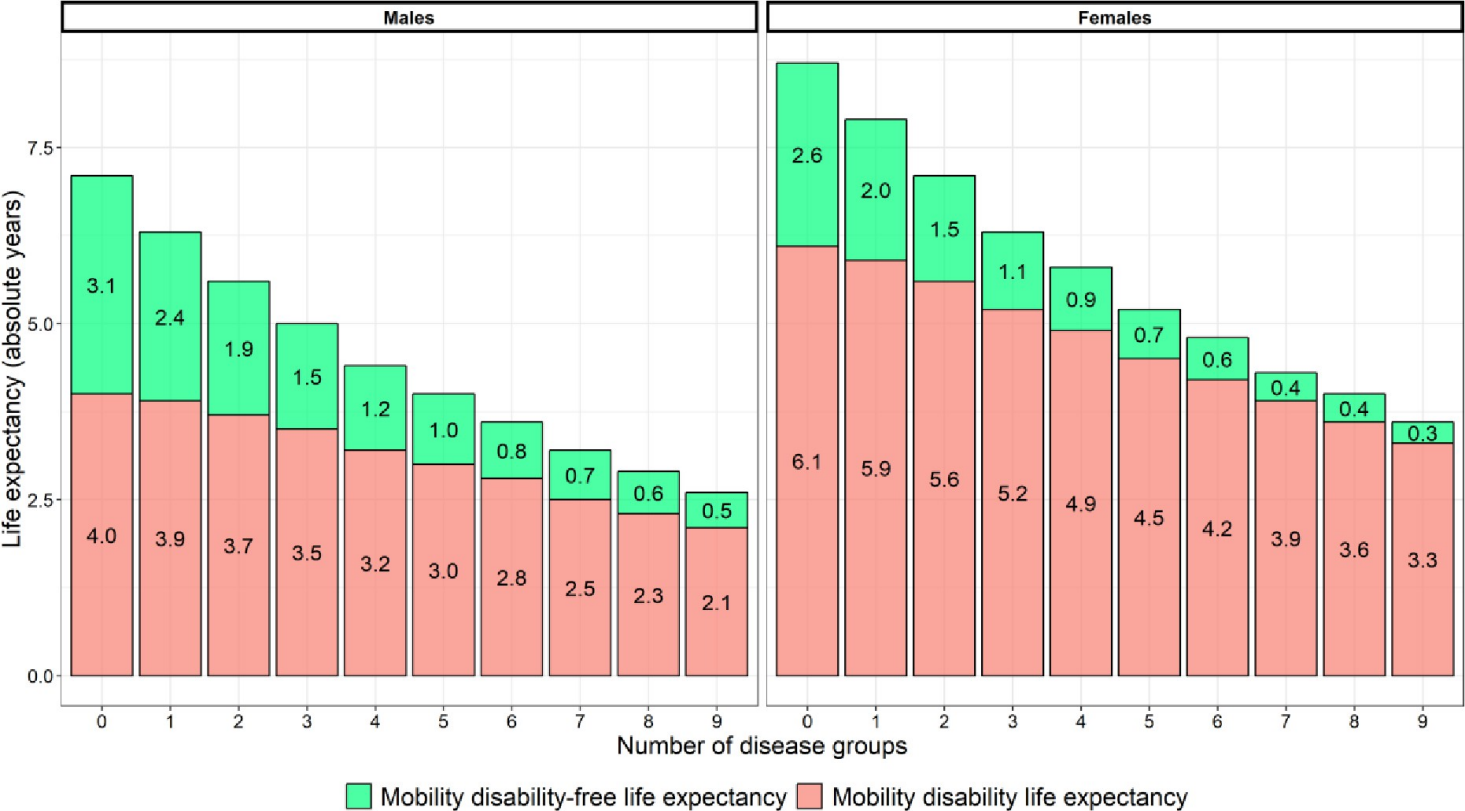
Remaining life expectancy (in years) spent with and without mobility disability for each disease group count, in male and female participants at age 85.

**Fig 4:**
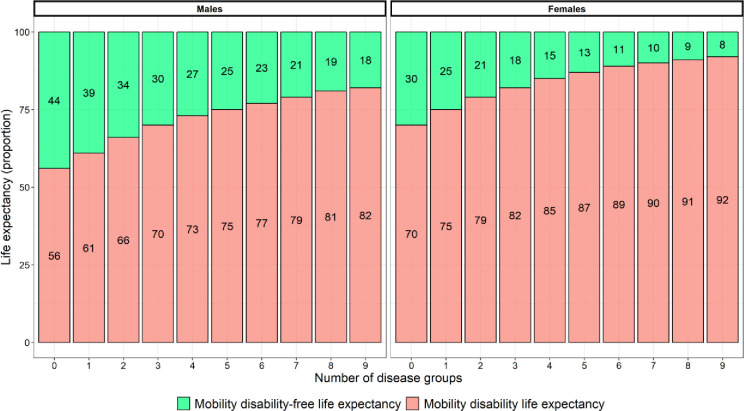
Remaining life expectancy (as a proportion) spent with and without mobility disability for each disease group count, in male and female participants at age 85.

In this study, increasing numbers of multiple long-term conditions were associated with a decrease in life expectancy (Fig 3) and an increase in the proportion of remaining time spent with mobility disability (Fig 4).

At age 85, males without disease have a remaining life expectancy of 7.1 years, 4.0 years of which are spent with mobility disability and 3.1 without mobility disability. Males with 1 diagnosed disease can expect to live 0.8 years less than males without disease (with their 6.3 years of remaining life comprising 3.9 years with and 2.4 years without mobility disability). Further increases in multiple long-term conditions followed a similar pattern, with fewer years of remaining life spent mobility disability free as the number of diseases increased. With 9 diagnosed diseases, 85-year-old males can, for example, expect to live 4.5 years less than males without disease (spending 2.1 of their remaining 2.6 years with mobility disability, and only 0.5 years without mobility disability, on average) (Fig 3). Confidence intervals for remaining life expectancy with and without mobility disability at each disease count can be found in Table 3.

The inverse association between increasing numbers of diseases and the decrease in the proportion of remaining time spent mobility disability free can be seen in Fig 4: Males without disease spend the greatest proportion of time mobility disability free (44%), and as the number of diseases increase this reduces, to 18% in males with 9 diseases.

**Fig 5:**
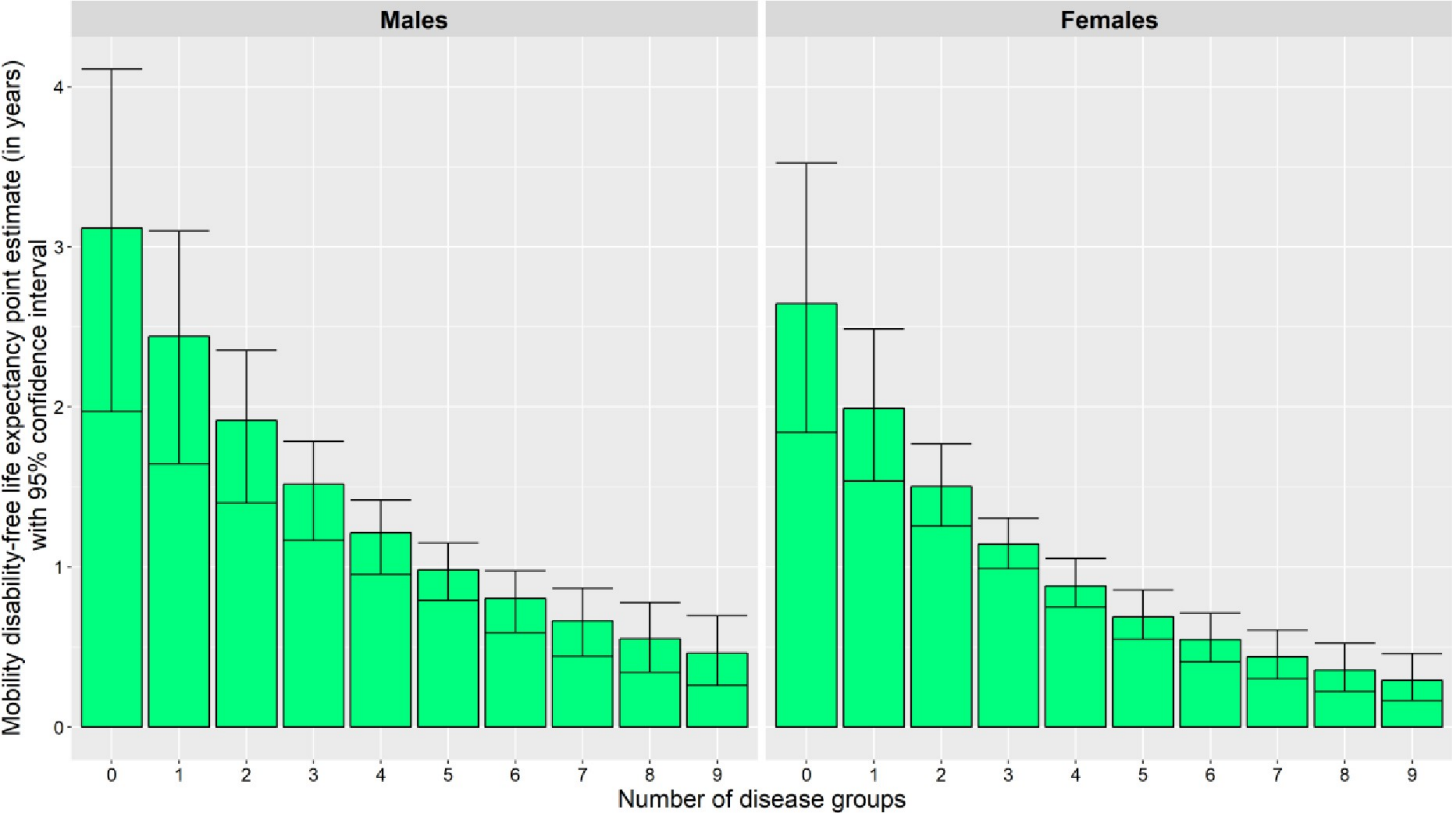
Graphical representation of point estimates with 95% confidence intervals for mobility disability-free life expectancy (in years) at each disease group count, in male and female participants at age 85.

For adjacent diseases, the relationship between the number of diseases and mobDFLE was not statistically significant. However, males with 3 diseases had a statistically significantly shorter (*p* < 0.05) mobDFLE than males without disease (1.5 years [95% CI: 1.2 to 1.8] compared to 3.1 years [95% CI: 2.0 to 4.1]); males with 5 diseases had a statistically significantly shorter (*p* < 0.05) mobDFLE than males with 3 diseases (1.0 years [95% CI: 0.8 to 1.1] compared to 1.5 years [95% CI: 1.2 to 1.8]), and males with 9 diseases had a statistically significantly shorter (*p* < 0.05) mobDFLE than males with 5 diseases (0.5 years [95% CI: 0.3 to 0.7] compared to 1.0 years [95% CI: 0.8 to 1.1]) (Table 3 and Fig 5).

**Table 3:** Point estimates with 95% confidence intervals for remaining life expectancy (in years) spent with and without mobility disability for each disease group count, in male and female participants at age 85.

	Males			Females		
Number of Disease Groups	mobDFLE^a^	mobDLE^b^	TLE^c^	mobDFLE^a^	mobDLE^b^	TLE^c^
None	3.1 (2.0–4.1)	4.0 (3.2–4.7)	7.1 (5.5–8.2)	2.6 (1.8–3.5)	6.1 (5.3–7.0)	8.7 (7.6–9.8)
1	2.4 (1.6–3.1)	3.9 (3.3–4.5)	6.3 (5.4–7.2)	2.0 (1.5–2.5)	5.9 (5.3–6.6)	7.9 (7.2–8.7)
2	1.9 (1.4–2.4)	3.7 (3.2–4.2)	5.6 (4.9–6.3)	1.5 (1.3–1.8)	5.6 (5.2–6.1)	7.1 (6.7–7.7)
3	1.5 (1.2–1.8)	3.5 (3.1–4.0)	5.0 (4.4–5.6)	1.1 (1.0–1.3)	5.3 (4.9–5.6)	6.4 (6.0–6.8)
4	1.2 (1.0–1.4)	3.2 (2.9–3.7)	4.4 (4.0–5.0)	0.9 (0.8–1.1)	4.9 (4.6–5.3)	5.8 (5.5–6.2)
5	1.0 (0.8–1.1)	3.0 (2.7–3.4)	4.0 (3.6–4.5)	0.7 (0.5–0.9)	4.6 (4.0.3–5)	5.2 (5.0–5.7)
6	0.8 (0.6–1.0)	2.8 (2.4–3.2)	3.6 (3.2–4.1)	0.5 (0.4–0.7)	4.2 (3.8–4.8)	4.8 (4.4–5.3)
7	0.7 (0.4–0.9)	2.5 (2.2–2.9)	3.2 (2.8–3.7)	0.4 (0.3–0.6)	3.9 (3.4–4.5)	4.3 (3.9–5.0)
8	0.6 (0.3–0.8)	2.3 (2.0–2.8)	2.9 (2.4–3.4)	0.4 (0.2–0.5)	3.6 (3.0–4.3)	3.9 (3.4–4.7)
9	0.5 (0.3–0.7)	2.1 (1.8–2.6)	2.6 (2.1–3.1)	0.3 (0.2–0.5)	3.3 (2.6–4.1)	3.5 (3.0–4.4)

^a^mobDFLE, mobility disability-free life expectancy.

^b^mobDLE, mobility disability life expectancy.

^c^TLE, total life expectancy.

A similar pattern prevailed for the female participants with 1 key difference: Multimorbidity was associated with mobility disability to a greater extent in females than males, yet females lived longer. At age 85, females without disease have a remaining life expectancy of 8.7 years: 6.1 years of which are spent with mobility disability and 2.6 without mobility disability. Females with 1 diagnosed disease can expect to live 0.8 years less than females without disease (with their 7.9 years of remaining life comprising 5.9 years with and 2.0 years without mobility disability). Further increases in multiple long-term conditions followed a similar pattern, with fewer years of remaining life spent mobility disability free as the number of diseases increased. With 9 diagnosed diseases, 85-year-old females can, for example, expect to live 5.1 years less than females without disease (spending 3.3 of their remaining 3.6 years with mobility disability, and only 0.3 years without mobility disability, on average) (Fig 3).

Females without any diseases therefore spent 30% of their remaining life mobility disability free, and as the number of diseases increased this proportion reduced, to 8% in females with 9 diseases (Fig 4).

Females with 2 diseases had a statistically significantly shorter (*p* < 0.05) mobDFLE than females without disease (1.5 years [95% CI: 1.3 to 1.8] compared to 2.6 years [95% CI: 1.8 to 3.5]); females with 4 diseases had a statistically significantly shorter (*p* < 0.05) mobDFLE than females with 2 diseases (0.9 years [95% CI: 0.8 to 1.1] compared to 1.5 years [95% CI: 1.3 to 1.8]), and females with 6 diseases had a statistically significantly shorter (*p* < 0.05) mobDFLE than females with 4 diseases (0.5 years [95% CI: 0.4 to 0.7] compared to 0.9 years [95% CI: 0.8 to 1.1]) (Table 3 and Fig 5).

## Discussion

To the best of our knowledge, our paper is the first to explore the association between multimorbidity and transitions in mobility disability in those aged ≥85, and to present estimates of mobDFLE at age 85 in the presence of multimorbidity. For every additional disease, the risk of incident mobility disability was increased, and the chance of recovery reduced. Female participants had a higher risk of incident mobility disability than the male participants, and a lower risk of death with mobility disability. Reductions in mobDFLE were observed with increasing numbers of multiple long-term conditions, and this association was more pronounced in the female participants.

Multimorbidity is the norm in those aged ≥85 [24] and is projected to increase [25]. Conceptual models of the disablement process place disease or active pathology at the start [26], and previous studies have shown that each additional chronic condition increases the risk of mobility disability [7,27]. Consistent with this, our analysis accounting for BMI and age suggests that the increasing prevalence of mild disability among older people is not just a consequence of population ageing and significant reversible factors contributing to multimorbidity such as obesity, as measured by BMI [28].

Previous studies have shown that continued reductions in mortality at older ages will result in more years with disability [29]. Attention is now focussing more on the quality of those extra years (healthy versus unhealthy life expectancy) [29]. To date, few studies have examined the effect of multimorbidity on life expectancy with and without disability [30,31], and none have examined its influence on mobDFLE in those aged ≥85. The reductions in mobDFLE that we observed with increasing numbers of multiple long-term conditions is therefore an interesting finding of our study. What is also apparent from previous research is the profound impact of mobility disability: It increases the risk of mortality, morbidity and hospital admission; self-care disability, social isolation and depression, a poorer quality of life and loss of independence [7,32,33]. It is also a risk factor for long-term care admission [7,32], yet most people would prefer to remain in their own homes as they age [34].

Regarding sex differences, females are known to live longer than males but with more disability [18]. This disability-survival paradox is still evident in people aged 85 years and over probably due to sex differences in the type and disabling impacts of diseases [18]; compared to males aged ≥85, females this age have a higher prevalence of long-term disabling conditions, such as arthritis, and a higher risk of incident disability from certain fatal conditions, like cerebrovascular disease [18]. Our observation that multimorbidity is disabling females more than males therefore extends previous research. Females aged ≥85 are also more likely to live alone through widowhood (Table 1) and therefore potentially manage mobility disability alone and have unmet needs in this regard [35], especially as informal care networks (e.g., children) are becoming more fragile for reasons including extended working life, greater female labour market participation and more geographically disparate families [36].

The strengths of our work include the long-term follow-up of a large sample of 85-year-olds, inclusive of those living in care homes, using an established measure of mobility disability [5,17]. Multiple long-term conditions were obtained from general practice medical records, as opposed to the less reliable method of self-report [13], and we accounted for pertinent confounding factors (for example, BMI) [37]. Multistate models also account for interval censored data, i.e., we know that transitions between mobility disability states took place between the study waves, based on multidimensional health assessment data, though not necessarily when. However, our work has limitations. It was beyond the scope of this work to examine the synergistic effects of specific combinations of diseases on mobility disability, but the literature highlights important disease pairs (such as arthritis and high blood pressure [38,39]). Furthermore, certain diseases may have had a stronger association with mobility disability than others. We might have missed episodes of intermittent disability and recovery of independence as mobility disability is a highly dynamic process in older people [40]. The possibility of residual and unmeasured confounding influencing our estimates also cannot be excluded. For example, the number of covariates that we could introduce was limited by the number of transitions; comparisons with available health assessment data show that rates of undiagnosed hypertension and ischaemic heart disease in the baseline sample were high [13], and we restricted multimorbidity to 9 disease groups, though the number of conditions included in studies of multimorbidity does vary widely [41]. Diseases were also grouped by body systems to increase power, and as has been the case elsewhere, we did not have information on disease severity [42]. In addition, we adjusted for education level instead of area-level deprivation [20], but the latter is the more complex measure. Loss to follow-up was primarily related to mortality [15], which we accounted for in our multistate model, but we were unable to account for other losses to follow-up that were assumed to be random. Finally, in terms of generalisability, there is little ethnic diversity in the Newcastle 85+ Study [13], so our results may not apply to non-white populations. In addition, future populations who go on to reach 85 years of age will have different disease profiles to those in our analytic sample (a 1912 birth cohort), as their earlier life experiences (and subsequent health trajectories) will be different: nonexposure to the First World War aftermath, for example. Other factors such as rising levels of multimorbidity [25] will also change the makeup of subsequent inception cohorts of 85-year-olds.

Our results suggest that there is no threshold beyond which multimorbidity becomes disabling in those aged ≥85; rather, each additional disease group is associated with a 16% increased risk of incident mobility disability. This translates to statistically significant reductions in mobDFLE at age 85, at several disease group cut points. Thus, multimorbidity (diagnoses in ≥2 disease groups for females and ≥3 for males) significantly shortens mobDFLE, and complex multimorbidity (diagnoses in ≥4 disease groups for females and ≥5 for males) reduces this even further. In terms of implications for practice, this reinforces calls for a greater focus on the prevention of multimorbidity [43] and further accrual of disease [25] as populations age. Approaches might include a primary care system that focuses on a multi, rather than single, disease paradigm, that promotes continuity of care [44], and reducing risk factor exposure (via smoking cessation and weight and blood pressure reduction, for example) from earlier in the life course [43].

More time spent with mobility disability could potentially lead to greater care needs and solutions for this will be required on several levels. Firstly, maintaining independence with increasing age should be a key focus for health/social care and reablement services [45]. Secondly, our results question whether an assessment of functional ability for older people with multimorbidity should become part of usual primary care practice, where the majority of multimorbidity management occurs, in order to proactively intervene in a timelier manner to maintain both health and independence [46,47]. Thirdly, the assessment and maintenance of physical function requires an integrated healthcare and social care approach [47].

The numbers of people aged ≥85 living with multimorbidity (≥2 conditions) and complex multimorbidity (≥4 conditions) in particular are also projected to increase [25]. Therefore, without interventions, we can infer that there will be more people aged 85 and over living with mobility disability in the coming years, so there is a need to consider the implications of this for future health and social service provision.

In terms of future research, we need to better understand the most common disease clusters, how can we stop diseases A, B and C from accruing, and potentially require the integration of single-condition clinical guidelines to help prevent conditions that a patient may not yet have but is at risk of developing in the future [48]. Targeting ageing hallmarks might be another way to prevent multimorbidity, and clinical trials are underway [49]. We also need a consensus definition of multimorbidity [41] in order to synthesise evidence about (a) the effects of different interventions for prevention and (b) predictive factors; this will help in the development of healthcare policy around the provision of preventative services [48]. Future research could also investigate whether (and at what age) multimorbidity becomes disabling in younger populations, including those of lower socioeconomic status, given the wide health inequalities that exist between rich and poor and the well-documented social patterning of multimorbidity, being more common and developing some 10 to 15 years earlier in deprived areas compared to affluent areas [50]. Finally, studies could examine the association between individual diseases and mobility disability, adjusting for residual disease count.

In summary, our findings based on an observational cohort study suggest that, in those aged ≥85, multimorbidity is an important determinant of mobility disability and the number of years spent living with it. The prevention, or postponement, of multimorbidity from earlier in the life course will thus have significant benefit to both the health and independence of people as they age, in addition to profound effects on their health and social care needs.

## Supporting information

S1 Appendix STROBE Statement—Checklist of items that should be included in reports of cohort studies. (DOCX)

S2 Appendix Recruitment and retention in the Newcastle 85+ Study. (DOCX)

S3 Appendix Disease group construction. (DOCX)

S4 Appendix Baseline sociodemographic and health characteristics of the Newcastle 85+ participants according to the number of disease groups. (DOCX)

## Abbreviations

BMI: body mass index
CI: confidence interval
ELECT: estimating life expectancies for continuous time
HR: hazard ratio
mobDFLE: mobility disability-free life expectancy
