# Supplementary figures and images for: The association between multimorbidity and mobility disability-free life expectancy in adults aged 85 years and over: A modelling study in the Newcastle 85+ cohort
Source: PLoS Med. 2022 Nov 14;19(11):e1004130. doi: 10.1371/journal.pmed.1004130 (PMC9662726; doi:10.1371/journal.pmed.1004130)

**S2 Appendix: Recruitment and retention in the Newcastle 85+ Study**


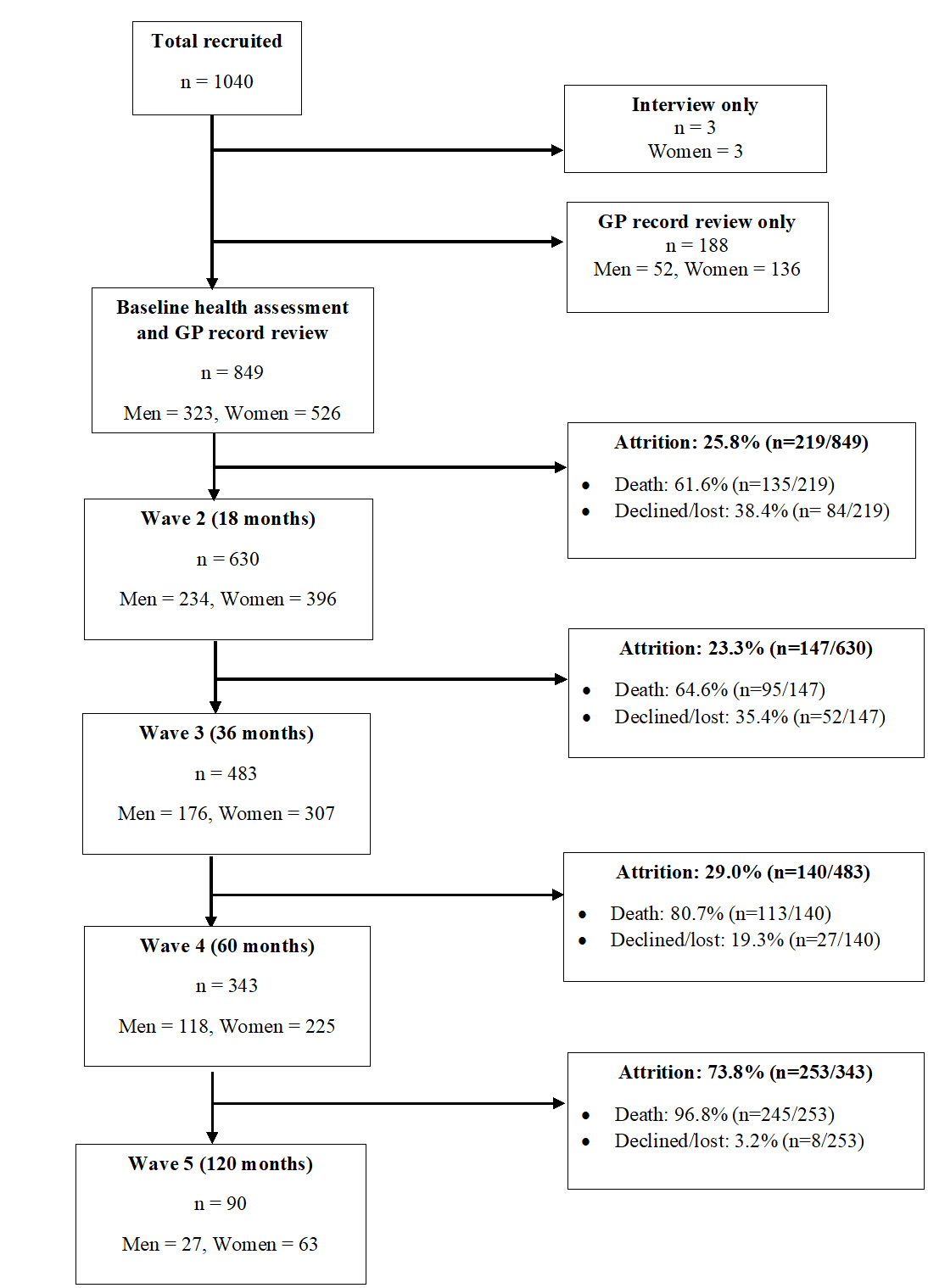

Supplement: S2 Appendix — (DOCX) [file pmed.1004130.s002.docx]
