# Supplementary material for: The association between multimorbidity and mobility disability-free life expectancy in adults aged 85 years and over: A modelling study in the Newcastle 85+ cohort
Source: PLoS Med. 2022 Nov 14;19(11):e1004130. doi: 10.1371/journal.pmed.1004130 (PMC9662726; doi:10.1371/journal.pmed.1004130)
Supplement: S3 Appendix — (DOCX) [file pmed.1004130.s003.docx]

**S3** Appendix: Disease group construction

| **Disease group** | **Composite make-up** |
| --- | --- |
| **Arthritis** | GP diagnosis of: generalised osteoarthritis, hand, hip and knee osteoarthritis, rheumatoid, degenerative / poly / gouty / septic / peri arthritis, lumbar spondylosis, cervical spondylosis, ankylosing spondylitis, psoriatic arthropathy |
| **Diabetes** | GP diagnosis of: Type I or II or type unspecified |
| **Hypertension** | GP diagnosis of hypertension |
| **Cardiac disease** | GP diagnosis of: Heart failure, angina, myocardial infarction, coronary artery bypass graft, coronary angioplasty / stent |
| **COPD** | GP diagnosis of:  Chronic bronchitis, emphysema, COPD |
| **Other respiratory disease** | GP diagnosis of: Bronchiectasis, pulmonary fibrosis, fibrosing alveolitis, asbestosis, pneumoconiosis, asthma |
| **Stroke** | GP diagnosis of stroke |
| **Other cerebrovascular disease** | GP diagnosis of: Transient ischaemic attack, carotid endarterectomy |
| **Cancer** | GP diagnosis of any cancer diagnosis in the previous 5 years excluding non-melanoma skin cancer |
