# Supplementary material for: The association between multimorbidity and mobility disability-free life expectancy in adults aged 85 years and over: A modelling study in the Newcastle 85+ cohort
Source: PLoS Med. 2022 Nov 14;19(11):e1004130. doi: 10.1371/journal.pmed.1004130 (PMC9662726; doi:10.1371/journal.pmed.1004130)
Supplement: S4 Appendix — (DOCX) [file pmed.1004130.s004.docx]

**S4 Appendix: Baseline sociodemographic and health characteristics of the Newcastle 85+ participants according to the number of disease groups**

|  | **0 disease groups,**  **% of total (n)** | **1 disease group,**  **% of total (n)** | **2-3 disease groups,**  **% of total (n)** | **≥4 disease groups,**  **% of total (n)** |
| --- | --- | --- | --- | --- |
| **Sex**  Male  Female | 42.0 (21)  58.0 (29) | 40.38 (42)  59.62 (62) | 36.76 (125)  63.24 (215) | 37.36 (130)  62.64 (218) |
| **Education (years) (mean (SD))** | 9.98 (1.85) | 10.12 (2.00) | 9.91 (1.89) | 9.84 (1.80) |
| **Housing**  Standard  Sheltered  Care home | 86.0 (43)  14.0 (7)  0.0 (0) | 74.04 (77)  15.38 (16)  10.58 (11) | 78.24 (266)  10.88 (37)  10.88 (37) | 74.43 (259)  15.23 (53)  10.34 (36) |
| **Living alone** | 59.18 (29) | 62.37 (58) | 61.39 (186) | 60.26 (188) |
| **Marital status**  Never married  Married  Divorced/separated  Widowed | 20.0 (10)  28.0 (14)  0.0 (0)  52.0 (26) | 7.77 (8)  34.95 (36)  0.97 (1)  56.31 (58) | 7.94 (27)  27.65 (94)  2.35 (8)  62.06 (211) | 6.96 (24)  31.59 (109)  4.06 (14)  57.39 (198) |
| **Deprivation (IMD)**  <25^th^ centile  25^th^-75^th^ centile  >75^th^ centile | 24.0 (12)  54.0 (27)  22.0 (11) | 22.12 (23)  46.15 (48)  31.73 (33) | 25.88 (88)  49.41 (168)  24.71 (84) | 25.57 (89)  52.01 (181)  22.41 (78) |
| **BMI (kg/m^2^)**  <18.5: underweight  18.5-24.99: healthy weight  25-29.99: overweight  >30: overweight and obese | 10.64 (5)  51.06 (24)  36.17 (17)  2.13 (1) | 8.79 (8)  60.44 (55)  24.18 (22)  6.59 (6) | 7.27 (21)  52.60 (152)  31.83 (92)  8.30 (24) | 4.45 (13)  46.92 (137)  35.96 (105)  12.67 (37) |
| **Mobility disability** | 30.0 (15) | 37.5 (39) | 55.29 (188) | 66.67 (232) |

SD = standard deviation; IMD = Index of Multiple Deprivation; BMI = body mass index

Where numbers do not add up to 842 data are missing
